# Patient satisfaction with outpatient physical therapy in Saudi Arabia

**DOI:** 10.1186/s12913-018-3646-0

**Published:** 2018-11-26

**Authors:** Ghadah Algudairi, Einas S. Al-Eisa, Ahmad H. Alghadir, Zaheen Ahmed Iqbal

**Affiliations:** 1 Physical Therapy Department, Security Forces Hospital, Riyadh, Kingdom of Saudi Arabia; 2 Rehabilitation Research Chair, College of Applied Medical Sciences, King Saud University, Riyadh, Kingdom of Saudi Arabia

**Keywords:** Patient satisfaction, Physical therapy, Saudi Arabia

## Abstract

**Background:**

Patient satisfaction (PS) is a key measure of the quality and outcome of healthcare systems which reflects patients’ experiences. The purpose of this study was to assess overall PS with outpatient physical therapy (PT) care in Saudi Arabia and identify associated characteristics and components.

**Methods:**

Four hundred patients who received PT treatment during 2017 were invited to participate in this study. The MedRisk Instrument for Measuring Patient Satisfaction with Physical Therapy Care (MRPS) was used to assess PS.

**Results:**

The average age of 358 (90%) respondents was 38.1 (SD 12.7) years, and a majority (77%) of them were female. At least 76% respondents reported feeling better after PT treatment, while the mean global satisfaction score of all respondents as per the MRPS was 3.56, indicating high satisfaction.

**Conclusion:**

PT is still at an early stage of development in Saudi Arabia and is an integral part of the healthcare sector. PS is the key to identify areas for improvement and provide high quality healthcare to the public.

## Background

Patient satisfaction (PS) is a multi-dimensional phenomenon that reflects the patient’s experiences while seeking healthcare [1]. It is directly associated with treatment outcomes and compliance with the treatment [2], and has been reported to be a key measure of quality and outcome of health care system [3, 4]. Patients who report higher satisfaction are often more likely to benefit from their treatment [5].

Outcome measures such as Physical Therapy Outpatients Survey (PTOPS) and the MedRisk instrument have been reported to be reliable and valid tools for measuring patient satisfaction with out-patient physical therapy services [6, 7]. Various studies conducted around the world, have reported PS with various forms of treatment, including medical management, surgery, and physical therapy [8, 9]. It has been related to various factors including patient age, their presenting condition, specific needs, expectations, previous experiences, social background, and personality [2, 4]. However, due to cultural differences, the findings of these studies cannot be generalized and applied to the rest of the world. To the best of our knowledge, there have been no studies conducted in Saudi Arabia that report PS with physical therapy treatment.

The purpose of this study was to assess overall PS, as well as its components with outpatient physical therapy care in Saudi Arabia, to identify associated patient characteristics. The findings of this study can provide guidance for hospital managers to improve service quality and PS.

## Methods

Four hundred patients who received physical therapy treatment for different conditions during 2017 were invited to participate in this study. The MedRisk Instrument for Measuring Patient Satisfaction with Physical Therapy Care (MRPS) was used to assess PS [7]. In addition, data on patients’ demographics (age, gender, educational status, occupation) and clinical characteristics (treatment type and duration, pain, recovery) were also collected. The questionnaire was uploaded online and its web link along with an explanation of the study’s purpose were sent to patients to invite them to participate in the study. The completion of the electronic survey was considered as consent for participation in the study. Further, a reminder email was sent to the patients 2 weeks after uploading the questionnaire. Ethical approval was obtained from the institutional review board of our university before data collection.

### Data analysis

The responses to the items and components of MRPS were coded on a 5-point Likert scale, with 5 indicating “complete satisfaction” and 1 indicating “complete non-satisfaction”. Data were presented as frequencies and percentages for categorical variables and mean and standard deviation (SD) for continuous variables. SPSS software (release 23.0, Armonk, NY: IBM Corp) was used to test any statistical differences between the variables. The overall MRPS scores were compared using non-parametric tests: Mann-Whitney U test for two-group variables and Kruskal-Wallis test for more than two-group variables. The difference was considered significant if *p* values were less than 0.05.

## Results

A total number of 358 (90%) patients completed the survey. The average age of respondents was 38.1 (SD 12.7) years, and a majority (77%) of them were female. The respondents reported that they sought physical therapy treatment for a variety of conditions, including musculoskeletal (67%) and neurological (17%) conditions. The affected body parts included the back (31%), lower limbs (27%), neck and shoulders (15%) and upper limbs (15%). At least 7% of the respondents reported they sought treatment for more than one body parts at a time. The average time spent by the majority of respondents (46%) was 16–30 min; at least 76% respondents reported feeling better after physical therapy treatment (Table 1).

**Table 1:** Demographic and clinical characteristics among respondents (*N* = 358)

	Number	Percentage
Age groups		
≤ 30	120	33.5%
31–50	187	52.2%
> 50	51	14.2%
Gender		
Male	83	23.2%
Female	275	76.8%
Educational status		
Less than secondary	50	14.0%
Secondary	80	22.3%
College or higher	228	63.7%
Occupation		
Working	149	41.6%
Student	64	17.9%
Retired	36	10.1%
Unemployed	109	30.4%
Body parts treated		
Neck and shoulder	54	15.3%
Back	108	30.5%
Upper limb	54	15.3%
Lower limb	94	26.6%
Multiple areas	25	7.1%
Others	19	5.4%
Time spent in the clinic (min)		
0–15 min	76	21.2%
16–30 min	165	46.1%
31–60 min	87	24.3%
> 60 min	30	8.4%
Pain duration in the body parts treated		
Last 3 months	87	24.3%
Last 6 months	52	14.5%
Last year	219	61.2%
Physical therapy treatment times during last year		
This is the first time	114	31.8%
2–4 times	127	35.5%
> 4 times	117	32.7%
Post treatment health status		
Better	272	76.0%
No change	61	17.0%
Worse	25	7.0%
Physical therapy speciality		
Musculoskeletal	237	66.9%
Neurology	60	16.9%
Sport injuries	25	7.1%
Women’s Health	16	4.5%
Others	16	4.5%
City of treatment		
Riyadh	263	80.9%
Outside Riyadh	62	19.1%

### PS with physical therapy treatment in Saudi Arabia

**Fig. 1:**
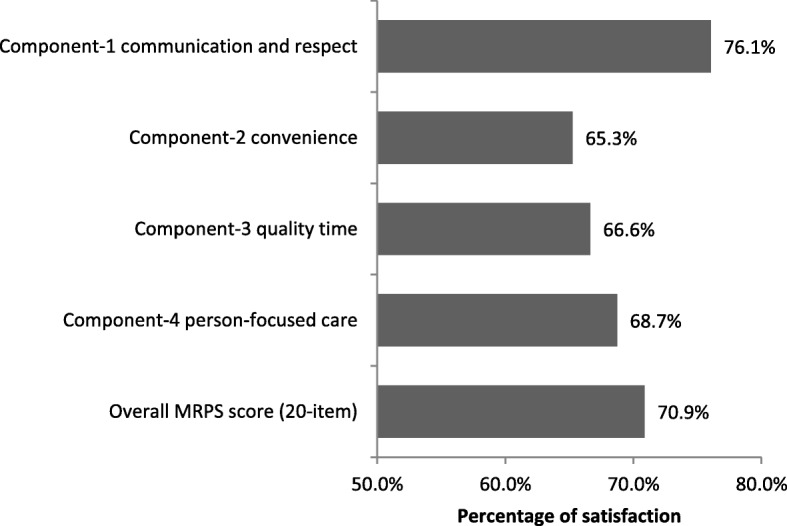
The percentage of components and overall scores of the MRPS among patient on physical therapy (*N* = 358)

On the 5-point Likert scale used, in which 5 indicates maximum satisfaction, the mean global satisfaction score of all respondents for item 19 (Overall, I am completely satisfied with the services I receive from my therapist) was 3.56 (SD 1.21). Figure 1 shows the mean percentage of scores for individual components of the MRPS. The mean satisfaction for individual items ranged from 4.15 for item 10 (My therapist treated me respectfully) to 2.94 for item 5 (This office provided convenient parking) (Table 2).

**Table 2:** Mean and SD of the items, components, and overall scores of the MRPS among respondents (*N* = 358)

	Number	Mean	SD
Component-1 Communication and respect	358	3.80	0.77
Item 1 The office receptionist was courteous	351	3.66	0.98
Item 9 My therapist thoroughly explained the treatment(s) I received	358	3.40	1.19
Item 10 My therapist treated me respectfully	358	4.15	0.85
Item 11 The office staff was respectful	352	4.05	0.89
Item 12 The therapist’s assistant/aide was respectful	328	3.95	0.88
Item 14 My therapist answered all my questions	358	3.75	1.04
Item 15 My therapist advised me how to avoid future problems	358	3.78	1.07
Item 18 My therapist gave me detailed home program instructions	358	3.75	1.10
Component-2 Convenience	358	3.26	0.64
Item 2 The registration process was appropriate	358	3.64	0.98
Item 3 The waiting area was comfortable	347	3.21	1.18
Item 4 The office location was convenient	358	2.95	1.22
Item 5 This office provided convenient parking	341	2.94	1.30
Item 6 I did not wait too long to see my therapist	358	3.27	1.16
Item 7 The office hours were convenient for me	358	3.56	1.06
Component-3 Quality time	358	3.33	0.91
Item 6 I did not wait too long to see my therapist	358	3.27	1.16
Item 8 My therapist spent enough time with me	358	3.21	1.25
Item 13 My therapist listened to my concerns	358	3.52	1.20
Component-4 Person-focused care	358	3.44	0.95
Item 3 The waiting area was comfortable	347	3.21	1.18
Item 16 The office and its facilities were clean	358	3.75	1.06
Item 17 The office used up-to-date equipment	358	3.35	1.19
Global items			
Item 19 Overall, I am completely satisfied with the services I receive from my therapist	358	3.56	1.21
Item 20 I would return to this office for future care	358	3.49	1.25
Overall MRPS score (20-item)	358	3.54	0.67

No significant differences were found in the mean satisfaction scores on the basis of the body area being treated. Female respondents reported more satisfaction for the ‘convenience’ component of MRPS (*p* < 0.01). A significant relationship was observed between the mean satisfaction scores and age, educational status, occupation, time spent in clinic, number of times physical therapy treatment was sought in the last year, post treatment health status and area of clinic where treatment was sought (*p* < 0.05) (Table 3).

**Table 3:** Comparison of average component and overall scores of the MRPS and demographic and clinical characteristics among respondents (*N* = 358)^a^

	Component-1 Communication and respect	Component-2 Convenience	Component-3 Quality time	Component-4 Person-focused care	Overall MRPS score (20-item)
Age groups					
< =30	3.90 ± 0.73	3.23 ± 0.65	3.47 ± 0.82	3.39 ± 0.96	3.58 ± 0.63
31–50	3.79 ± 0.80	3.31 ± 0.64	3.31 ± 0.97	3.51 ± 0.93	3.56 ± 0.70
> 50	3.64 ± 0.72	3.17 ± 0.61	3.08 ± 0.85	3.29 ± 0.95	3.40 ± 0.64
*P-value*	0.05	0.218	**0.029**	0.227	0.128
Gender					
Male	3.71 ± 0.87	3.02 ± 0.74	3.39 ± 0.93	3.26 ± 1.09	3.41 ± 0.78
Female	3.83 ± 0.73	3.34 ± 0.59	3.32 ± 0.91	3.49 ± 0.89	3.58 ± 0.63
*P-value*	0.637	**0.001**	0.423	0.153	0.22
Educational status					
Less than secondary	3.99 ± 0.65	3.36 ± 0.58	3.06 ± 0.93	3.71 ± 0.94	3.67 ± 0.58
Secondary	3.88 ± 0.74	3.36 ± 0.58	3.26 ± 0.93	3.66 ± 0.78	3.63 ± 0.62
College or higher	3.73 ± 0.79	3.21 ± 0.67	3.42 ± 0.89	3.30 ± 0.98	3.48 ± 0.70
*P-value*	0.053	0.182	**0.024**	**0.001**	0.134
Occupation					
Working	3.69 ± 0.83	3.19 ± 0.65	3.37 ± 0.93	3.25 ± 0.98	3.46 ± 0.73
Student	3.82 ± 0.76	3.09 ± 0.67	3.49 ± 0.80	3.26 ± 0.93	3.48 ± 0.64
Retired	3.74 ± 0.81	3.30 ± 0.63	3.36 ± 0.99	3.74 ± 0.64	3.57 ± 0.66
Unemployed	3.97 ± 0.64	3.45 ± 0.59	3.17 ± 0.90	3.69 ± 0.92	3.69 ± 0.60
*P-value*	0.183	**0.003**	0.089	**< 0.001**	0.104
Body parts treated					
Neck and shoulder	3.89 ± 0.69	3.39 ± 0.50	3.44 ± 0.86	3.50 ± 0.81	3.64 ± 0.57
Back	3.80 ± 0.86	3.32 ± 0.64	3.37 ± 0.92	3.38 ± 1.00	3.57 ± 0.71
Upper limb	3.76 ± 0.81	3.21 ± 0.68	3.42 ± 0.87	3.32 ± 1.10	3.49 ± 0.73
Lower limb	3.72 ± 0.74	3.14 ± 0.72	3.15 ± 0.89	3.47 ± 0.95	3.46 ± 0.69
Multiple areas	4.13 ± 0.53	3.38 ± 0.53	3.43 ± 1.25	3.63 ± 0.82	3.73 ± 0.60
Others	3.67 ± 0.65	3.15 ± 0.63	3.39 ± 0.71	3.49 ± 0.66	3.45 ± 0.53
*P-value*	0.101	0.312	0.328	0.851	0.3
Time spent in the clinic (min)					
0–15 min	3.55 ± 0.80	3.20 ± 0.61	3.01 ± 0.91	3.28 ± 0.95	3.33 ± 0.67
16–30 min	3.80 ± 0.78	3.25 ± 0.63	3.41 ± 0.87	3.43 ± 0.92	3.56 ± 0.68
31–60 min	3.98 ± 0.65	3.34 ± 0.68	3.35 ± 0.91	3.55 ± 0.96	3.67 ± 0.61
> 60 min	3.93 ± 0.76	3.24 ± 0.68	3.63 ± 0.99	3.53 ± 0.97	3.66 ± 0.69
*P-value*	**0.003**	0.341	**0.006**	0.341	**0.012**
Pain duration in the body parts treated					
Last 3 months	3.92 ± 0.72	3.25 ± 0.63	3.44 ± 1.01	3.56 ± 0.92	3.64 ± 0.67
Last 6 months	3.74 ± 0.91	3.16 ± 0.76	3.16 ± 1.00	3.29 ± 1.06	3.44 ± 0.78
Last year	3.77 ± 0.74	3.29 ± 0.62	3.33 ± 0.84	3.42 ± 0.92	3.53 ± 0.64
*P-value*	0.183	0.546	0.189	0.343	0.154
Physical therapy treatment times during last year					
This is the first time	3.79 ± 0.84	3.25 ± 0.68	3.54 ± 0.94	3.36 ± 1.00	3.56 ± 0.72
2–4 times	3.83 ± 0.72	3.26 ± 0.57	3.19 ± 0.88	3.44 ± 0.88	3.54 ± 0.63
> 4 times	3.78 ± 0.74	3.28 ± 0.69	3.28 ± 0.88	3.50 ± 0.96	3.53 ± 0.67
*P-value*	0.687	0.855	**0.005**	0.559	0.731
Post treatment health status					
Better	4.01 ± 0.60	3.36 ± 0.61	3.46 ± 0.88	3.62 ± 0.86	3.73 ± 0.55
No change	3.25 ± 0.83	3.00 ± 0.62	2.87 ± 0.88	2.93 ± 0.91	3.04 ± 0.65
Worse	2.87 ± 0.88	2.91 ± 0.78	3.03 ± 0.89	2.65 ± 1.13	2.76 ± 0.77
*P-value*	**< 0.001**	**< 0.001**	**< 0.001**	**< 0.001**	**< 0.001**
Physical therapy specialty					
Musculoskeletal	3.79 ± 0.75	3.30 ± 0.64	3.35 ± 0.95	3.43 ± 0.95	3.55 ± 0.67
Neurology	3.92 ± 0.76	3.23 ± 0.61	3.43 ± 0.85	3.48 ± 0.88	3.63 ± 0.63
Sport injuries	3.78 ± 0.80	2.99 ± 0.82	3.16 ± 0.64	3.28 ± 1.10	3.40 ± 0.74
Women’s Health	3.51 ± 0.97	3.21 ± 0.55	3.10 ± 0.78	3.27 ± 0.85	3.35 ± 0.67
Others	3.80 ± 0.88	3.24 ± 0.61	3.21 ± 1.03	3.50 ± 1.05	3.52 ± 0.73
*P-value*	0.529	0.31	0.436	0.819	0.43
City of treatment					
Riyadh	3.86 ± 0.78	3.29 ± 0.67	3.37 ± 0.92	3.57 ± 0.88	3.60 ± 0.68
Outside Riyadh	3.58 ± 0.77	3.11 ± 0.52	3.33 ± 0.94	2.97 ± 1.05	3.33 ± 0.68
*P-value*	**0.01**	**0.012**	0.947	**< 0.001**	**0.005**

^a^Data are presented as means and standard deviations, Non-parametric tests were used; Mann-Whitney test for two-group variables and Kruskal Wallis test for more than two-group variables

Significant *p*-values (bold) indicate that the component and overall scores are different between the categories of the variable

### Relation between individual components of MRPS and global satisfaction score

Table 4 shows the correlations of different components of the MRPS with its global items [10, 11] among respondents. All components (Communication and respect, Convenience, Quality time, and Person-focused care) significantly correlated with global satisfaction scores (*p* < 0.001).

**Table 4:** Correlations of different components of the MRPS with its global items [10, 11] among respondents (*N* = 358)

	Item 19 Overall, I am completely satisfied with the services I receive from my therapist		Item 20 I would return to this office for future care	
	Spearman Correlation Coefficient	*p*-value*	Spearman Correlation Coefficient	*p*-value*
Component-1 Communication and respect	0.79	*< 0.001*	0.74	*< 0.001*
Component-2 Convenience	0.50	*< 0.001*	0.50	*< 0.001*
Component-3 Quality time	0.43	*< 0.001*	0.40	*< 0.001*
Component-4 Person-focused care	0.61	*< 0.001*	0.60	*< 0.001*

* significant

## Discussion

Hospitals in Saudi Arabia have recently been adopting different ways to ensure better health care for patients and meet accreditation standards [12]. For this purpose, it is necessary to have knowledge of patients’ attitudes towards the quality of service provided by hospitals. PS has been reported to be a key outcome measure for assessing the quality and efficacy of hospital care [13, 14]. Despite the emphasis, no such research has been conducted to explore patients’ perceptions related to physical therapy in the region. The present study aimed to determine PS with physical therapy treatment in Saudi Arabia. At least 76% respondents reported feeling better after seeking physical therapy treatment, while the mean global satisfaction score of all respondents according to the MRPS was 3.56, indicating a high level of satisfaction.

MRPS has been widely used to report patient satisfaction with physical therapy treatment around the world [15]. Studies conducted in Brazil and Australia have reported high patient satisfaction with physiotherapeutic care (with mean score of 4.50 and 4.55 respectively) [11, 16]. A patient is satisfied if their needs are fulfilled, and they have been provided with adequate information about their condition and treatment; hence, PS represents one aspect of treatment success [7]. In addition to the care outcome, patients’ needs also relate to the quality of the treatment process [17]. Various studies have demonstrated that PS plays a role in patients’ compliance with medical advice, follow up and even improvement in health status [10, 18, 19]. Therefore, PS with physical therapy is fast emerging as an outcome variable of critical importance [5].

Our results show high satisfaction among patients seeking physical therapy treatment in Saudi Arabia, irrespective of the nature of their condition and the body parts involved. Similar results were reported in studies conducted in various clinical settings across America, UK, Australia, and Europe, indicating high-quality care from physical therapy management across the world [4, 11, 20–24]. On the other hand, the overall satisfaction with PT was positively correlated with all components of PS, including communication and respect, convenience, quality time, and personal care. Perceptions about the quality of care are influenced by provider and patient interaction as well as the positive attitude of the therapist, in addition to their technical competence. Professionals who are warm and friendly generate higher levels of patients’ satisfaction [3, 16].

Different studies from around the world have demonstrated higher levels of PS in the management of lower back pain with physical therapists than physicians. This is due to the interest in patients shown by physical therapists, shorter waiting time, and the actual time spent with the clinician and in counselling [8, 25]. Physical therapists’ friendly attitude, helpfulness, listening to the patients’ concerns, and their understanding nature makes them more popular among patients [26].

Our results show that PS is associated with various factors including patients’ gender, age, educational status, occupation, time spent in clinic, history of physical therapy treatment, outcome of treatment, and area of clinic where treatment was sought. Previous studies have also reported that patients’ degree of satisfaction depends on various characteristics, including their race and gender [6, 27]. Female patients reported higher satisfaction with physical therapy as compared to male patients [4, 26]. Patients who reported seeking physical therapy for acute musculoskeletal conditions reported higher satisfaction than those who sought physical therapy for chronic conditions [22, 28]. Additionally, there is some evidence that suggests older patients are more satisfied with physical therapy care [4, 26].

Study has shown that although there was moderate awareness about physical therapy among physicians in Saudi Arabia, referral of patients for physical therapy treatment depends on their specialty and work experience [29]. Other factors reported to affect PS also include the type of patient referring system [5]. Patients reported higher satisfaction when they received physical therapy through direct access instead of referral through physician [3, 30]. People who received care through prepaid group practices reported to be less satisfied than those who received treatment through fee-for-service practice [6].

Previous studies have also linked PS with the type of hospital where treatment was sought [3]. In some countries, such as the USA, university hospitals are considered to provide better quality of care than other private and government hospitals [31]. Similarly, our study was also conducted in a university hospital showing higher patient satisfaction.

Healthcare access in Saudi Arabia has improved dramatically over the past three decades [12]. However, respondents from outside the capital city had lower satisfaction scores than those in the capital region. This difference may be due to cultural factors, or regional differences in health practices within a country [32]. In Saudi Arabia, the attitude of patients’ and therapists’ in rural regions differ from those in urban regions [33]. In order to generalize the findings, it is suggested that similar studies should be conducted in all regions of the country, and covering all types of hospitals and clinics.

### Limitations

A self-report questionnaire was used for data collection, which increases the chances of respondents’ over- or under-estimating their experiences. Positive response bias may also have affected our results. Participation in this study could have been limited as the present questionnaire based study and those who were not interested or didn’t have time did not respond. This study could be repeated among a larger sample of patients representative of different parts of the country.

## Conclusion

A key health service policy is currently being implemented in Saudi Arabia to adopt various methods for improving the quality of health care, and apply these methods across all health sectors to ensure an appropriate level of efficiency [12]. Our study indicates that knowledge about PS is necessary to identify areas that need improvement, and enable the availability of high-quality healthcare services to the public. Physical therapy is still at an early stage of development and documentation in Saudi Arabia [34]. As it is an integral player in the health care sector, patient feedback can be used systematically in order to improve health care.
